# Multidisciplinary approach to occlusal rehabilitation in a patient with true hemifacial hyperplasia and temporomandibular joint ankylosis: a case report

**DOI:** 10.1186/s12903-024-05307-1

**Published:** 2024-12-20

**Authors:** Eun-Hack Andrew Choi, Jun-Hyeong Bae, Seeyoon Lee, Jee Hwan Kim, Loi Phuoc Nguyen, Jun-Young Kim, Sung-Hwan Choi

**Affiliations:** 1 Department of Orthodontics, Institute of Craniofacial Deformity, Yonsei University College of Dentistry, 50-1 Yonsei-Ro, Seodaemun-Gu, Seoul, 03722 Republic of Korea; 2 Department of Prosthodontics, Yonsei University College of Dentistry, 50-1 Yonsei-Ro, Seodaemun-Gu, Seoul, 03722 Republic of Korea; 3 Department of Maxillofacial Surgery, University of Medicine and Pharmacy at Ho Chi Minh City, Ho Chi Minh, Vietnam; 4 Department of Oral and Maxillofacial Surgery, Yonsei University College of Dentistry, 50-1 Yonsei-Ro, Seodaemun-Gu, Seoul, 03722 Republic of Korea

**Keywords:** True hemifacial hyperplasia, Temporomandibular joint ankylosis, Multidisciplinary approach, Gap arthroplasty, Three-dimensional simulation

## Abstract

**Background:**

This case report details a true hemifacial hyperplasia with temporomandibular joint ankylosis case managed through a multidisciplinary approach involving department of oral and maxillofacial surgery, orthodontics, and prosthodontics.

**Case presentation:**

A 42-year-old female patient presented with a chief complaint of limited mouth opening. Clinically, the patient exhibited severe facial asymmetry due to hyperplasia of the left facial region. Cone-beam computed tomography findings revealed overgrowth of the left mandible, zygomatic bone, and maxillary bone including alveolar bone, along with bony ankylosis of the temporomandibular joint. To alleviate the restricted mouth opening, gap arthroplasty was initially performed. Additionally, alveoloplasty was carried out to address occlusal interference caused by the overgrown alveolar bone. Orthodontic treatment was conducted to reduce mandibular molar width and achieve proper overjet. For reconstruction of the left side occlusion, dental implants were placed using an implant surgical guide, followed by prosthetic rehabilitation. Total treatment duration was 48 months, resulting in stabilization of the patient’s occlusion.

**Conclusion:**

By setting feasible goals through consultations among specialists from each department, based on three-dimensional simulations, successful and efficient occlusal rehabilitation can be achieved in a true hemifacial hyperplasia patient.

## Background

Hemifacial hyperplasia (HFH) is a congenital malformation characterized by prominent unilateral overdevelopment of hard and soft tissues of the face. It is known as a rare condition with an estimated prevalence of 1 in 86,000 live births [1, 2]. HFH is typically detected at birth. It progresses until puberty, although it is generally believed to remain unchanged throughout an individual’s lifetime. It has been classified as true HFH and partial HFH by Rowe [1]. True HFH is defined as unilateral viscerocranial enlargement involving all components, including both soft and hard tissues. When the enlargement is limited to a single structure, it is defined as partial HFH.

In most cases, the enlargement and resulting facial asymmetry do not worsen in adulthood. Treatment is usually not necessary unless for cosmetic purposes [3, 4]. However, various interventions including surgical procedures are required if there are functional issues. Especially if bony enlargements and ankyloses to the temporomandibular joint (TMJ) occur, aggressive treatment is necessary to maintain function. This may involve condylectomy, arthroplasty, and various orthognathic procedures to correct asymmetry [3].

Most patients with HFH exhibit macrodontia on the affected side, along with alveolar bone growth and enlargement of the affected side of the tongue [5–8]. This can cause secondary tooth movement, making orthodontic treatment necessary for occlusal rehabilitation. If the prognosis of the teeth is poor or if there are limitations to orthodontic movement, appropriate prosthetic approaches are also needed. Thus, multidisciplinary approach is needed for total occlusal rehabilitation in HFH.

This case report details a case of true HFH with TMJ ankylosis managed through a multidisciplinary approach involving departments of oral and maxillofacial surgery, orthodontics, and prosthodontics.

## Case presentation

### Diagnosis and etiology

A 42-year-old female patient presented to the department of oral and maxillofacial surgery with a chief complaint of restricted mouth opening for the past six months. From birth to 2 years old, there was no apparent asymmetry, although the skin on the left cheek was reported to be redder than that on the right cheek. From age 3–4, noticeable asymmetry developed visually, which progressively worsened until the age of 19, after which it ceased to progress. After the asymmetry stopped worsening, the patient was advised by a local clinic to undergo orthognathic two-jaw surgery. However, the patient opted to undergo only maxillary surgery about 20 years ago. Six months ago, symptoms of jaw locking and clicking sounds worsened, leading to restricted mouth opening, although no further visual asymmetry was observed. The patient has had a history of migraines and tinnitus, with tinnitus occurring more frequently during the period when TMJ symptoms worsen.

**Fig. 1:**
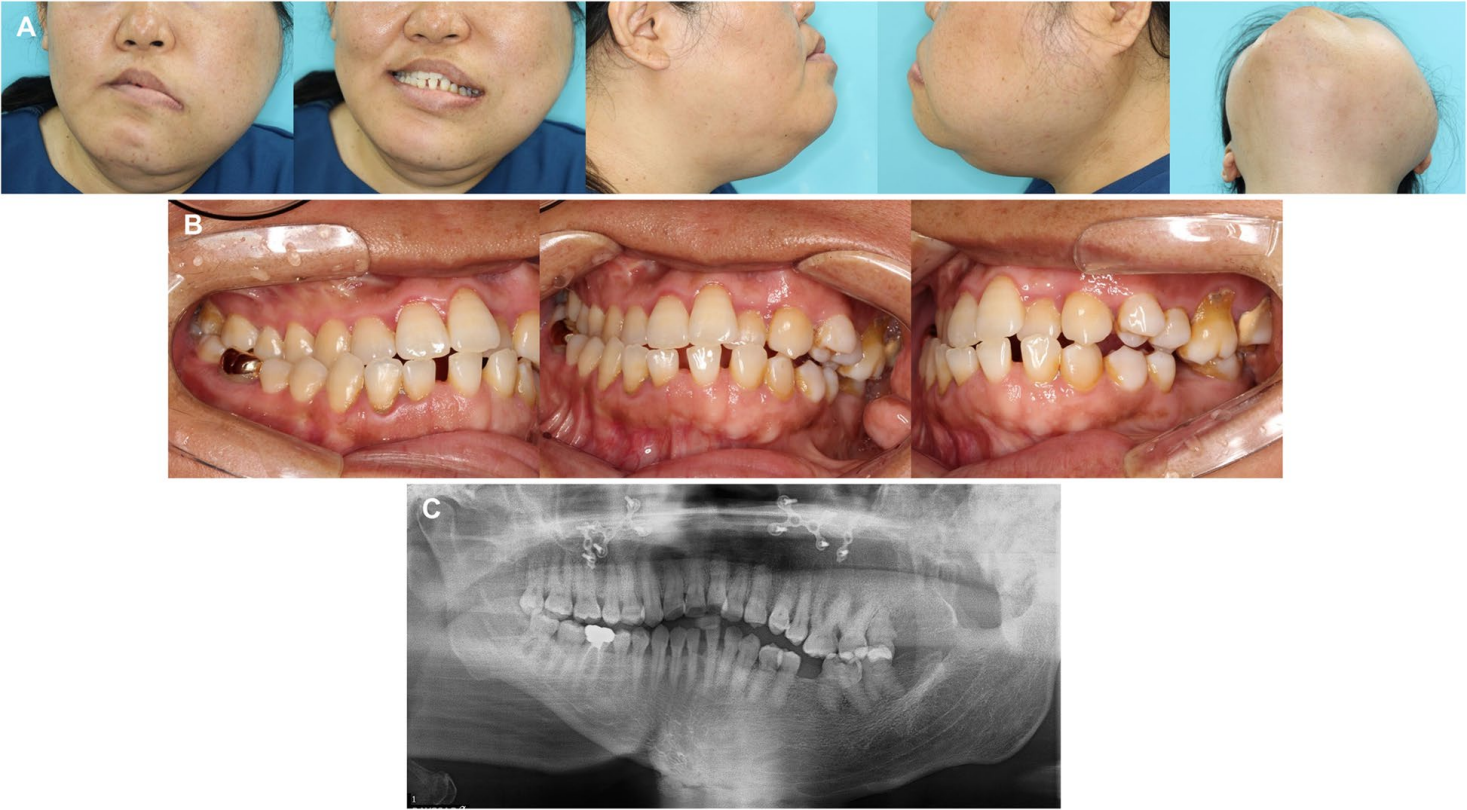
Pretreatment records. **A** Extraoral photographs. Hypertrophy of the left facial region is observed, accompanied by menton deviation to the right side and facial asymmetry with occlusal canting. **B** Intraoral photographs. Crossbite and Class III molar relationship are observed on the left side, while scissor bite in the premolar region and Class II molar relationship are observed on the right side. **C** Panoramic radiographs. Hypertrophy of the left mandible, including the condylar process and coronoid process, is observed, as well as macrodontia in the left posterior region

Extraoral examination revealed extensive facial hypertrophy on the left side, accompanied by significant asymmetry, canting of the occlusal plane, downward displacement of the left corner of the mouth, and rightward deviation of maxillary and mandibular dental midlines (Fig. 1A). Intraoral findings showed a crossbite on the right side, a scissor bite on the left side, spacing in the mandibular anterior region, missing mandibular first molars, and overeruption of maxillary first molars (Fig. 1B). Panoramic radiographs revealed an overall hypertrophic left mandible, severe hypertrophy of the left condyle, and overeruption of the left maxillary molars (Fig. 1C).

**Fig. 2:**
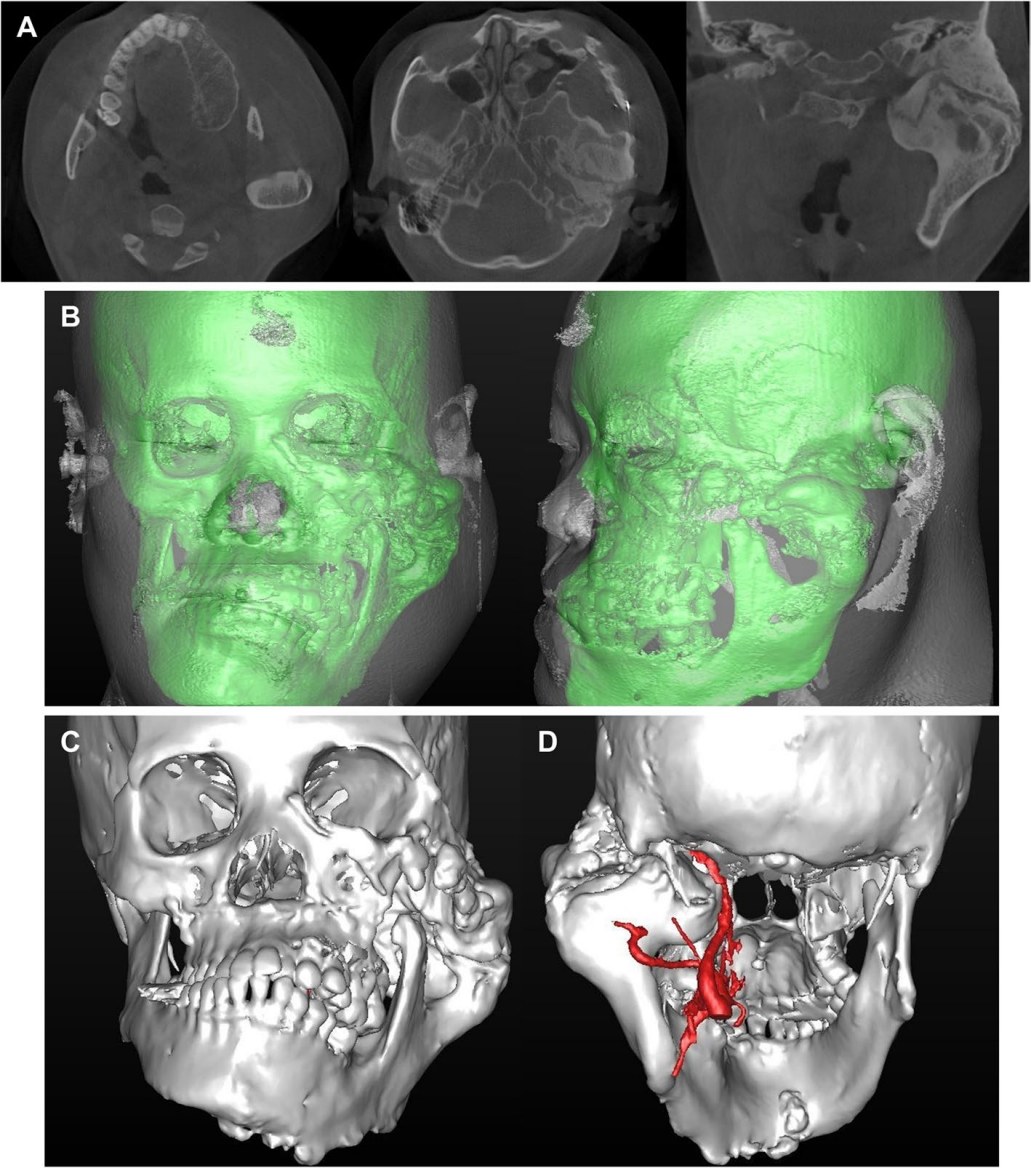
Pretreatment CBCT images and 3D reconstruction. **A** Axial and coronal CBCT images, **B** 3D reconstruction of hard and soft tissues, **C** 3D reconstruction of hard tissue only, **D** 3D reconstruction of carotid artery using angiography

Cone-beam computed tomography (CBCT) images indicated hyperplasia of the left maxilla, mandible, zygomatic bone, and alveolar bone, particularly showing irregular bone formation and partial ankylosis between the hypertrophic condyle and the articular fossa (Fig. 2A). Three-dimensional (3D) reconstruction of CBCT images confirmed bone proliferation starting from the lower border of the left orbit and significant inferomedial displacement of the left mandible due to condylar hyperplasia (Fig. 2B, C).

### Treatment objectives and plan

The primary objective was to restore oral function rather than achieving ideal occlusion. Detailed treatment goals were as follows: 1) to resolve mouth opening limitation, and 2) to restore masticatory function by establishing proper molar occlusion in the left side region. Initially, gap arthroplasty was planned to address the mouth opening limitation. Given the significant hypertrophy of the left maxillary alveolar bone and overeruption posterior teeth as well as partial occlusal contact with the mandibular posterior teeth, it was anticipated that the mandible would not return to its normal position after arthroplasty. Therefore, it was planned to reassess and perform maxillary alveoloplasty following arthroplasty. Orthodontically, the treatment plan included resolving the scissor bite and crossbite, securing normal posterior overjet, adjusting the vertical position of the left maxillary posterior teeth, resolving the mandibular anterior spacing, and creating adequate space for implant restoration of the left first molar.

### Treatment progress

**Fig. 3:**
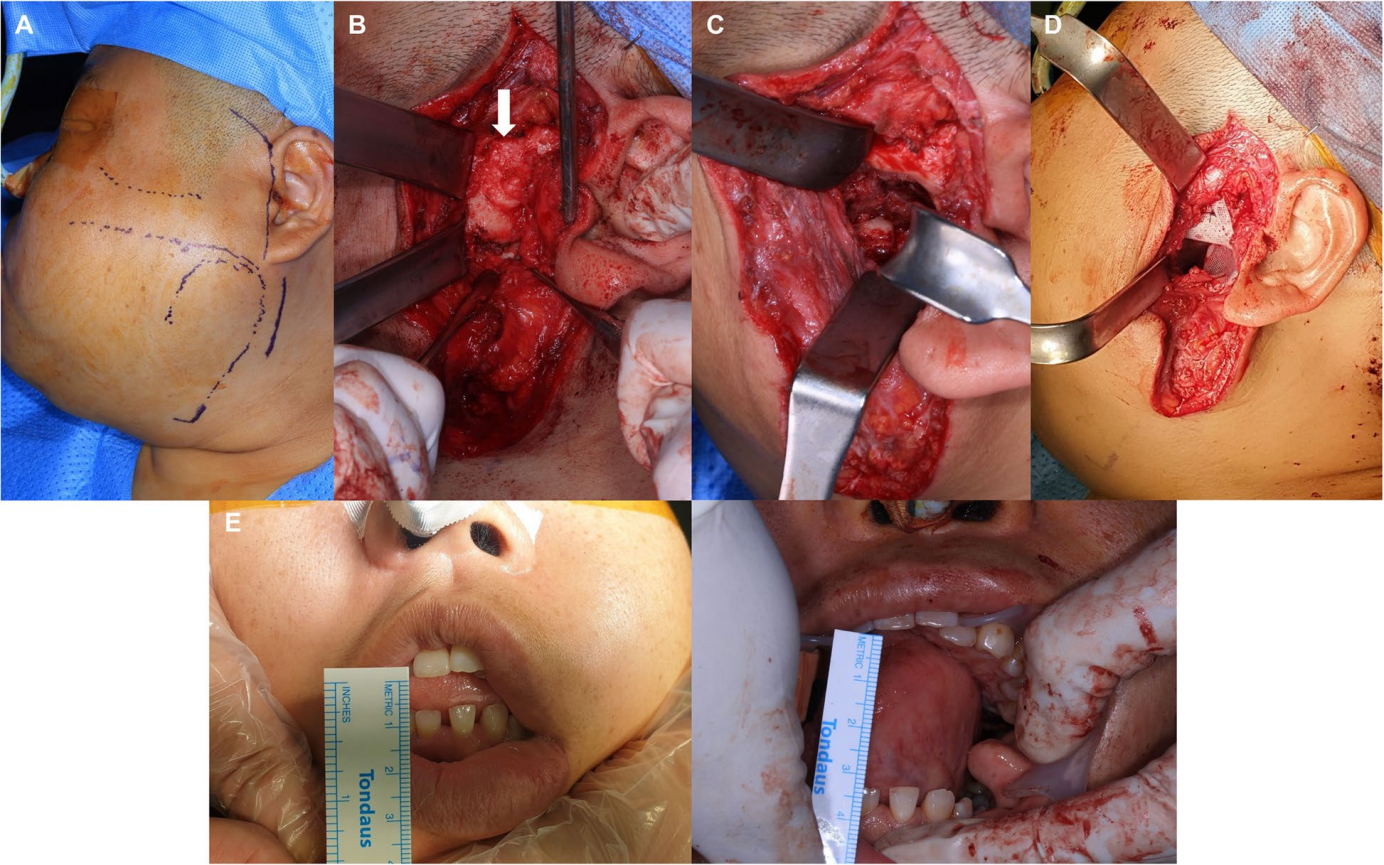
Procedure of arthroplasty. **A** Preoperative incision lines, **B** Detection of the glenoid fossa and ankylosed condylar head, **C** Removal of ankylosed bone, **D** Insertion of silastic sheath, **E** Preoperative and postoperative maximum mouth opening assessments

Prior to performing arthroplasty, 3D reconstruction via angiography was conducted to confirm the anatomical position and displacement of the carotid artery (Fig. 2D). Due to the limitation in mouth opening, general endotracheal anesthesia was administered using a nasotracheal tube. Additional local anesthesia was administered to the left preauricular area, followed by preauricular and submandibular skin incisions (Fig. 3A). During dissection and flap elevation, the buccal branch of the facial nerve was detected and preserved. The glenoid fossa, condylar head, and neck were identified, confirming the presence of ankylosis (Fig. 3B). Gap arthroplasty was then performed using an osteotome, a reciprocating saw, an oscillating saw, and a sagittal saw (Fig. 3C). To prevent postoperative re-ankylosis, a silastic sheath was inserted and 4 cc of Evicel® (fibrin sealant) was applied (Fig. 3D). The surgical procedure resulted in a maximum mouth opening of 35 mm (Fig. 3E). Additionally, maxillary left second and third molars and mandibular left third molar, which had poor prognosis due to periodontal disease and caries, were extracted.

Postoperatively, to prevent re-ankylosis, early active physiotherapy was initiated under the supervision of the surgical team. Active and passive jaw-opening exercises were started within 48 h after surgery. Patients were instructed to perform repeated maximum mouth-opening exercises using depressors (tongue blades) under the supervision of the surgeon during hospital visits. These exercises were carried out multiple times daily, with a focus on maintaining the surgical gap and progressively increasing the mouth-opening distance.

**Fig. 4:**
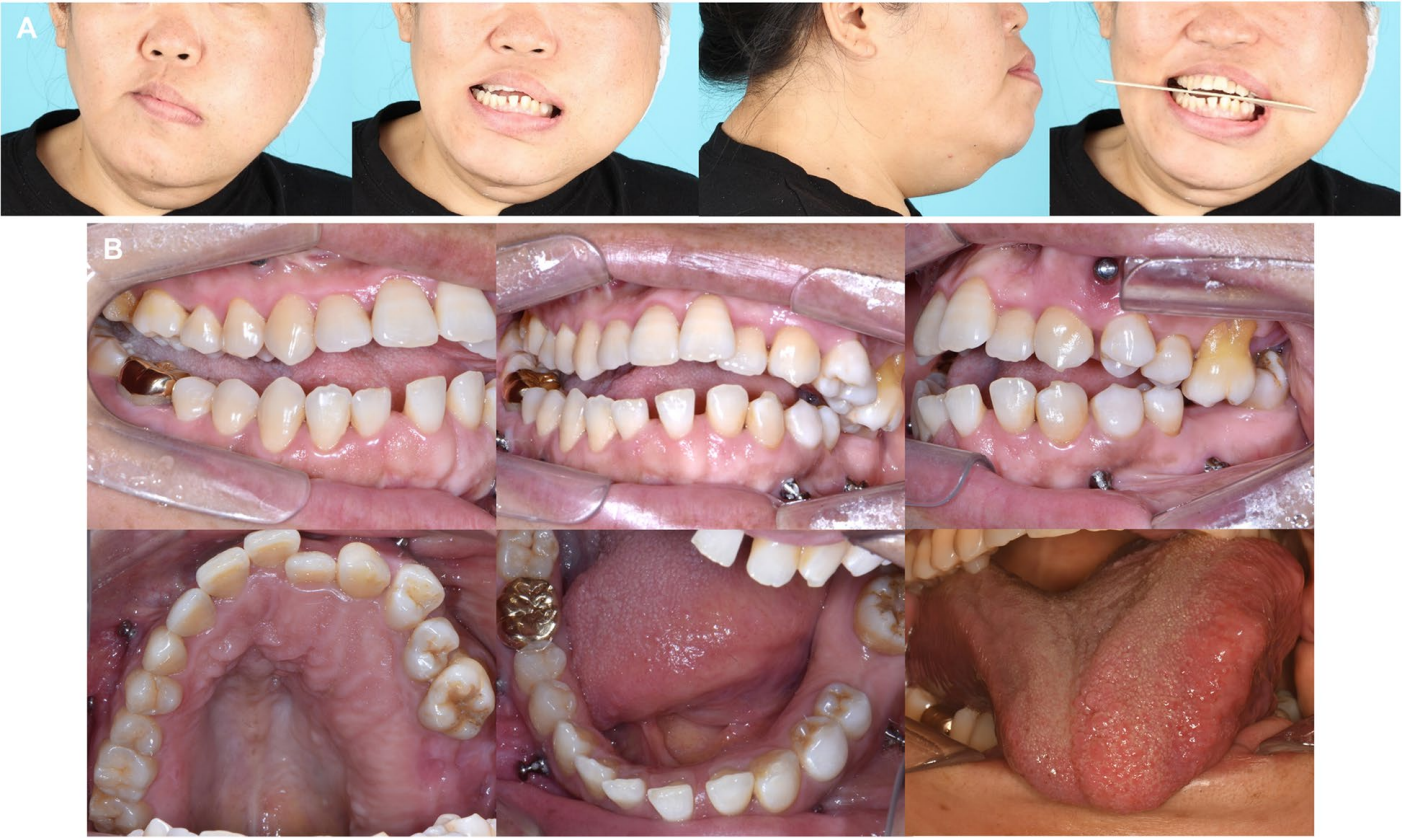
One-month post-arthroplasty photographs. **A** Extraoral photographs, **B** Intraoral photographs and tongue photographs

One month after arthroplasty, the patient visited the orthodontic department. Extraoral photographs showed a slight reduction in the volume of condylar area tissues. However, residual facial asymmetry with inferomedial rotation of the mandible and canting of the occlusal plane remained (Fig. 4A). Intraoral examination revealed generalized anterior and posterior open bite on the right side due to premature contact between the maxillary left tuberosity and the mandibular retromolar area. Overeruption of the maxillary left first molar into the mandibular left alveolar bone was observed (Fig. 4B). A remarkable left-sided enlargement of the tongue was also noted.

**Fig. 5:**
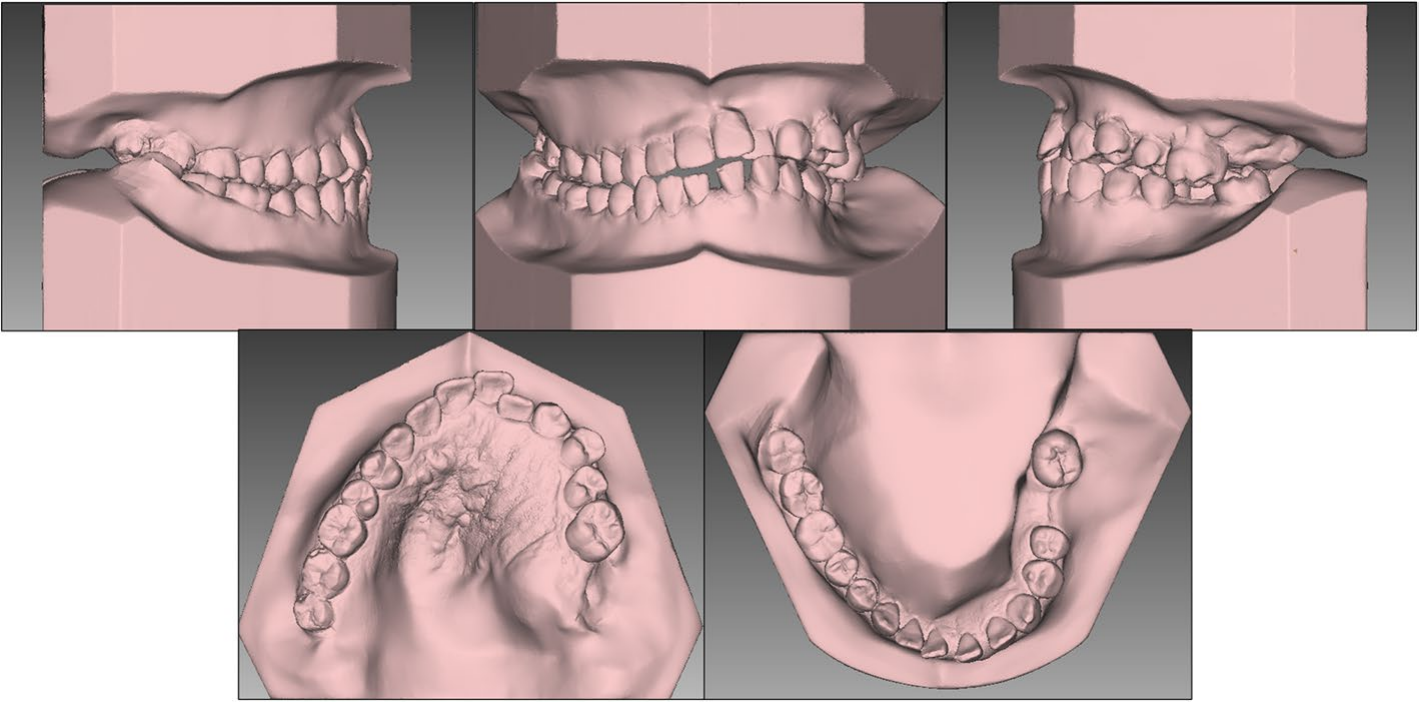
One-month post-arthroplasty dental casts

 Macrodontia was observed from left maxillary and mandibular canines to the molars, with buccolingual, mesiodistal width and height being approximately 1–2 mm larger than those of the teeth on the opposite side (Fig. 5).

**Fig. 6:**
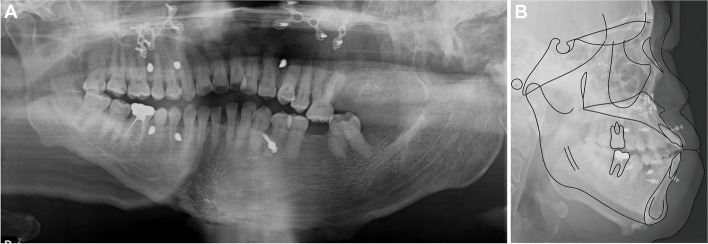
One-month post-arthroplasty radiographs. **A** Panoramic radiographs, **B** Lateral cephalogram and tracing

Panoramic radiographs confirmed the removal of the ankylosed condylar head (Fig. 6A). The lateral cephalogram and tracing, along with the measurements, are presented in Fig. 6B and Table 1.

**Table 1:** Cephalometric analysis

Measurements	Pretreatment	Posttreatment
SNA (°)	87.8	87.8
SNB (°)	86.8	87.7
ANB (°)	1.0	0.1
Wits (mm)	-6.3	-5.3
SN-GoMe (°)	33.5	32.9
U1 to SN (°)	127.0	123.3
L1 to GoGn (°)	84.9	90.2
Upper lip to E-line (mm)	7.1	4.8
Lower lip to E-line (mm)	10.0	8.7

**Fig. 7:**
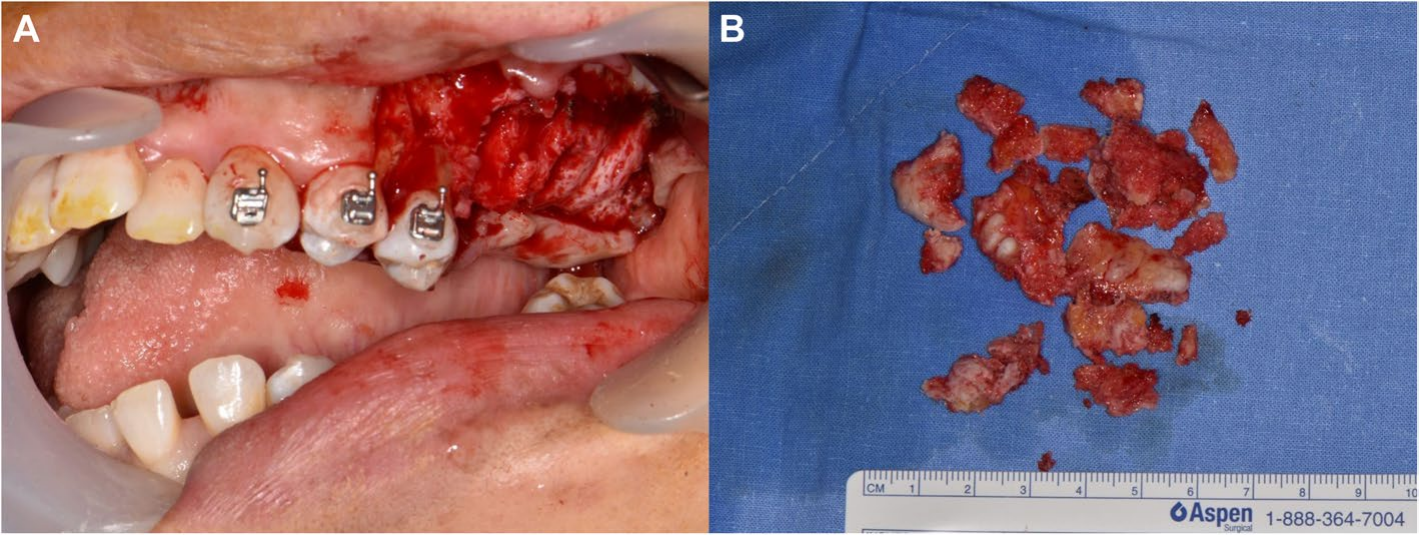
Procedure of alveoloplasty of the maxilla. **A** Immediate postoperative intraoral photograph, **B** Removed bone fragment

Nine months after arthroplasty, maxillary osteoplasty, including alveoloplasty of the left maxilla, was performed to eliminate premature contact areas (Fig. 7A, B). Maxillary left first molar showed poor periodontal prognosis with gingival recession. Maxillary left first and second premolars exhibited early contact with the mandibular teeth post-tuberosity osteoplasty, necessitating extraction.

**Fig. 8:**
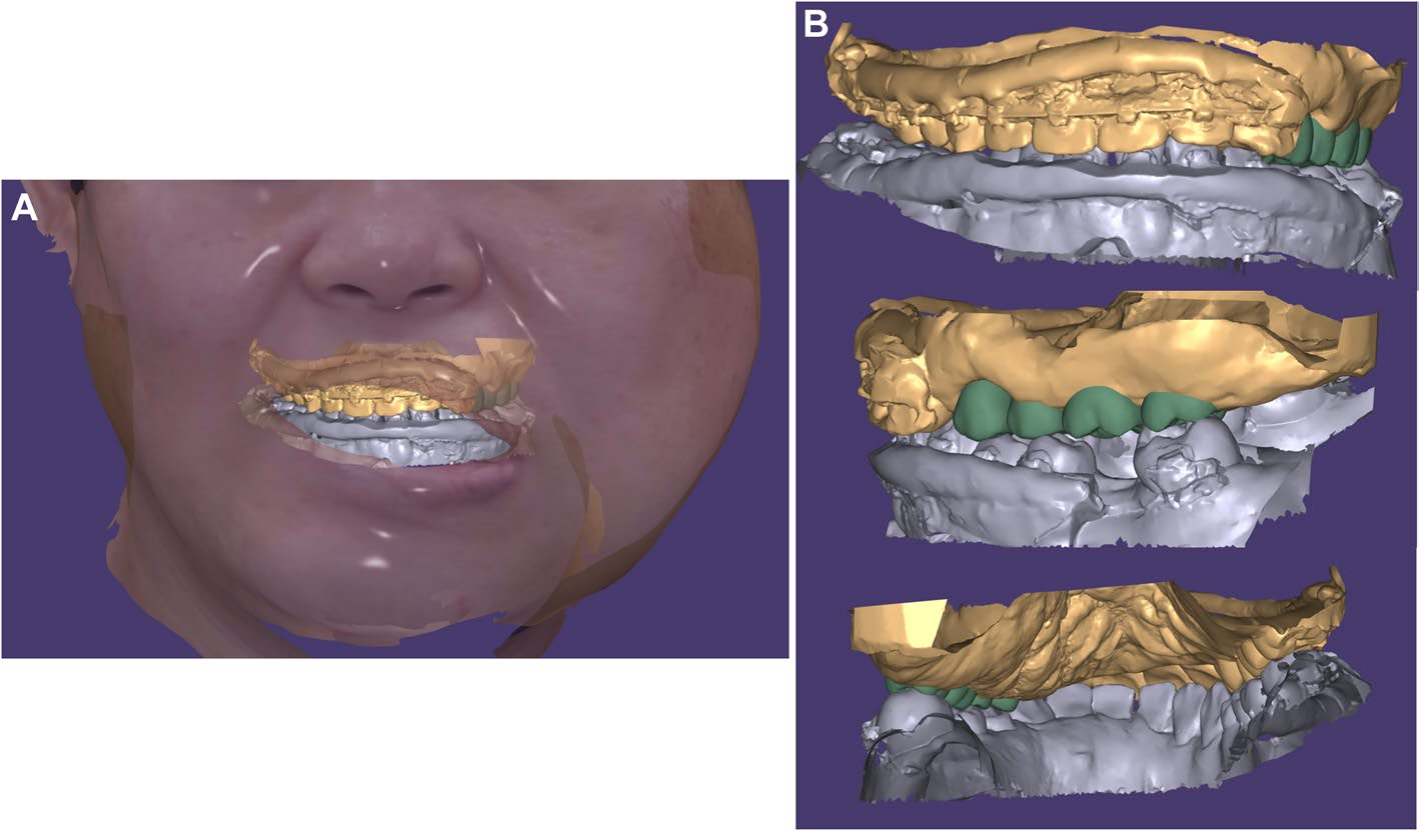
3D Simulation of implant prosthesis. **A** Images merged with CBCT and facial and intraoral scans, **B** Front, right side, and rear views of implant prosthesis simulation

After surgery, orthodontic appliances were placed on the maxillary and mandibular teeth for leveling, alignment, and space closure in the mandibular anterior region, along with creating space for implant placement of the mandibular left first molar. In the case of the left mandibular posterior region, there was no reference for vertical or horizontal positioning of the teeth due to the absence of opposing teeth. Therefore, 12 months after starting orthodontic treatment, we simulated the final prosthesis to set goals for necessary tooth movements in specific directions. After aligning facial and intraoral scan files with the CBCT, implant prostheses were virtually positioned (Fig. 8A). Vertically, the right maxillary posterior teeth were used as a reference to prevent canting of the occlusal plane. Mesiodistally and buccopalatally, positions were set to ensure suitable bone quality for implantation, canine and molar key, and arch harmony (Fig. 8B). Based on simulation results and subsequent discussion among orthodontic, oral and maxillofacial surgery, and prosthodontic departments, the following plans were made: 1) additional alveoloplasty of the maxilla to secure 7 mm of vertical dimension, 2) 2–3 mm palatal movement of the left maxillary anterior teeth, and 3) 3 mm additional posterior movement of the left mandibular second molar with lingual crown torque.

**Fig. 9:**
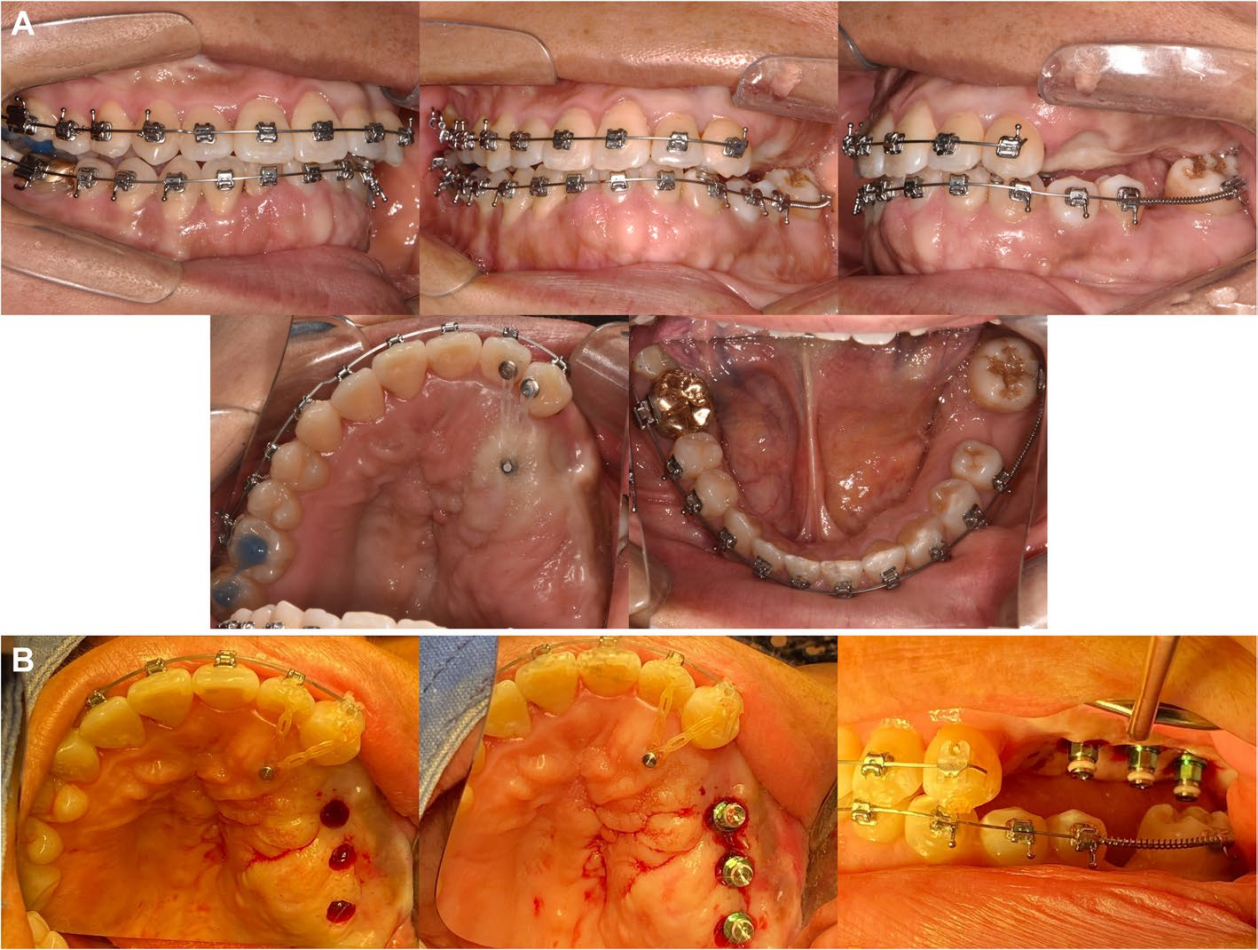
Intraoral photographs during orthodontic treatment and the 1st implant surgery. **A** After 13 months of orthodontic treatment, **B** First implant surgery after 16 months of orthodontic treatment

After 13 months of orthodontic treatment, 0.016 $\times$ 0.022-inch beta titanium wires were engaged in both arches (Fig. 9A). Miniscrew was placed on the palate to apply orthodontic forces to move the buccally positioned maxillary left lateral incisor and canine palatally. Palatal crown torque was applied on the wire. An open coil spring was activated to distalize the mandibular second molar and lingual crown torque was applied.

After 16 months of orthodontic treatment, additional alveoloplasty of the maxillary left posterior region was performed to secure vertical dimension. An implant of 4.5 mm in diameter and 10 mm in length was then placed in the position of the second premolar. Implants of 5.0 mm in diameter and 10 mm in length (Osstem, Seoul, South Korea) were placed in positions of the first and second molars (Fig. 9B).

**Fig. 10:**
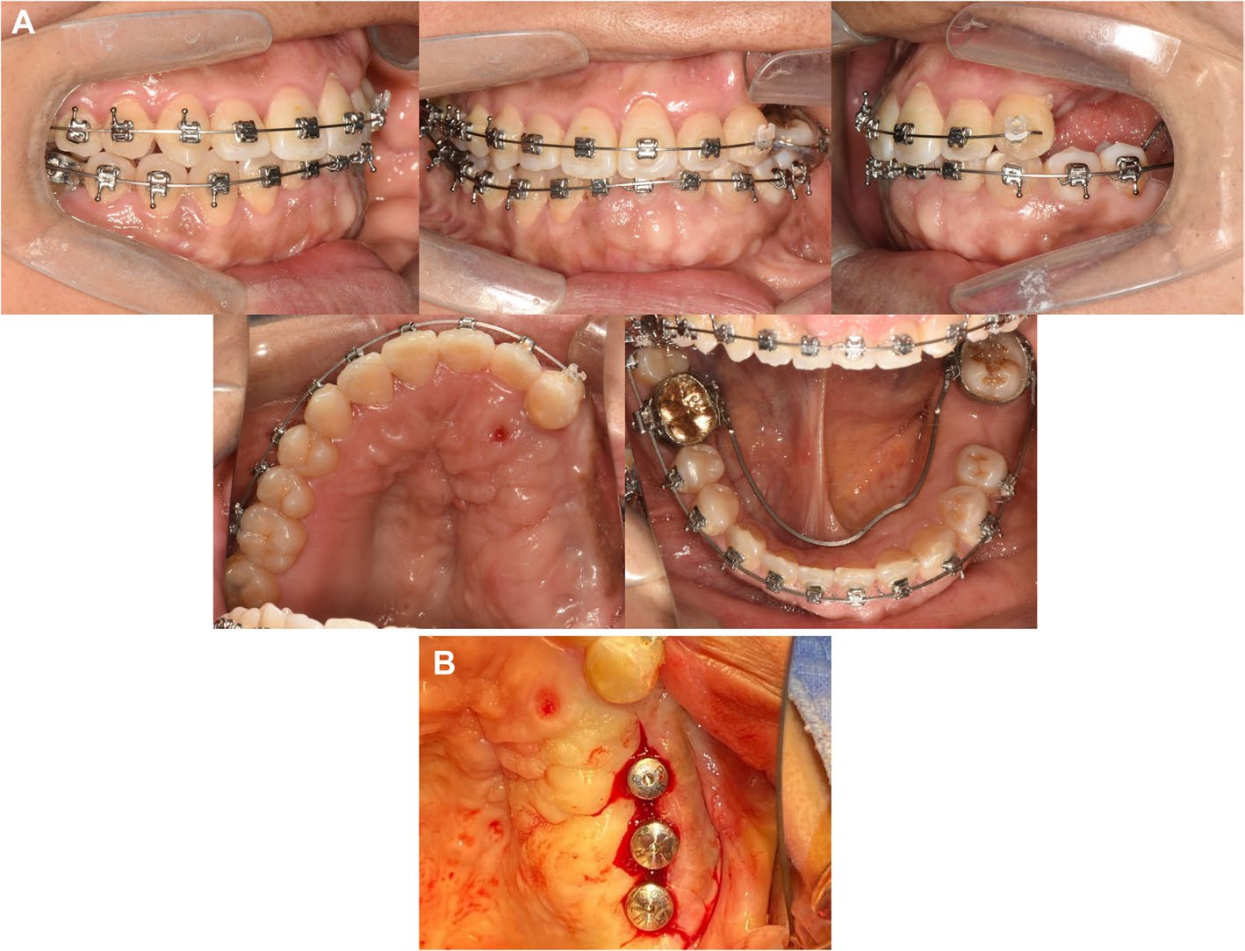
Intraoral photographs during orthodontic treatment and 2nd implant surgery. **A** After 20 months of orthodontic treatment, **B** Second implant surgery after 20 months of orthodontic treatment

After 20 months of orthodontic treatment, a lingual arch was placed for additional torque control of the mandibular left second molar (Fig. 10A). The 2nd implant surgery was completed (Fig. 10B).

At 28 months into orthodontic treatment, the 1st surgery for the mandibular left first molar implant was performed, followed by the 2nd implant surgery at 32 months. The patient wanted to maintain the implant restoration space with an orthodontic appliance. Therefore, at 34 months into orthodontic treatment, final implant prostheses were placed in both the maxilla and mandible and orthodontic appliances were removed. Fixed lingual retainers were bonded to the anterior teeth and circumferential retainers were used for both the maxilla and the mandible.

### Treatment results

**Fig. 11:**
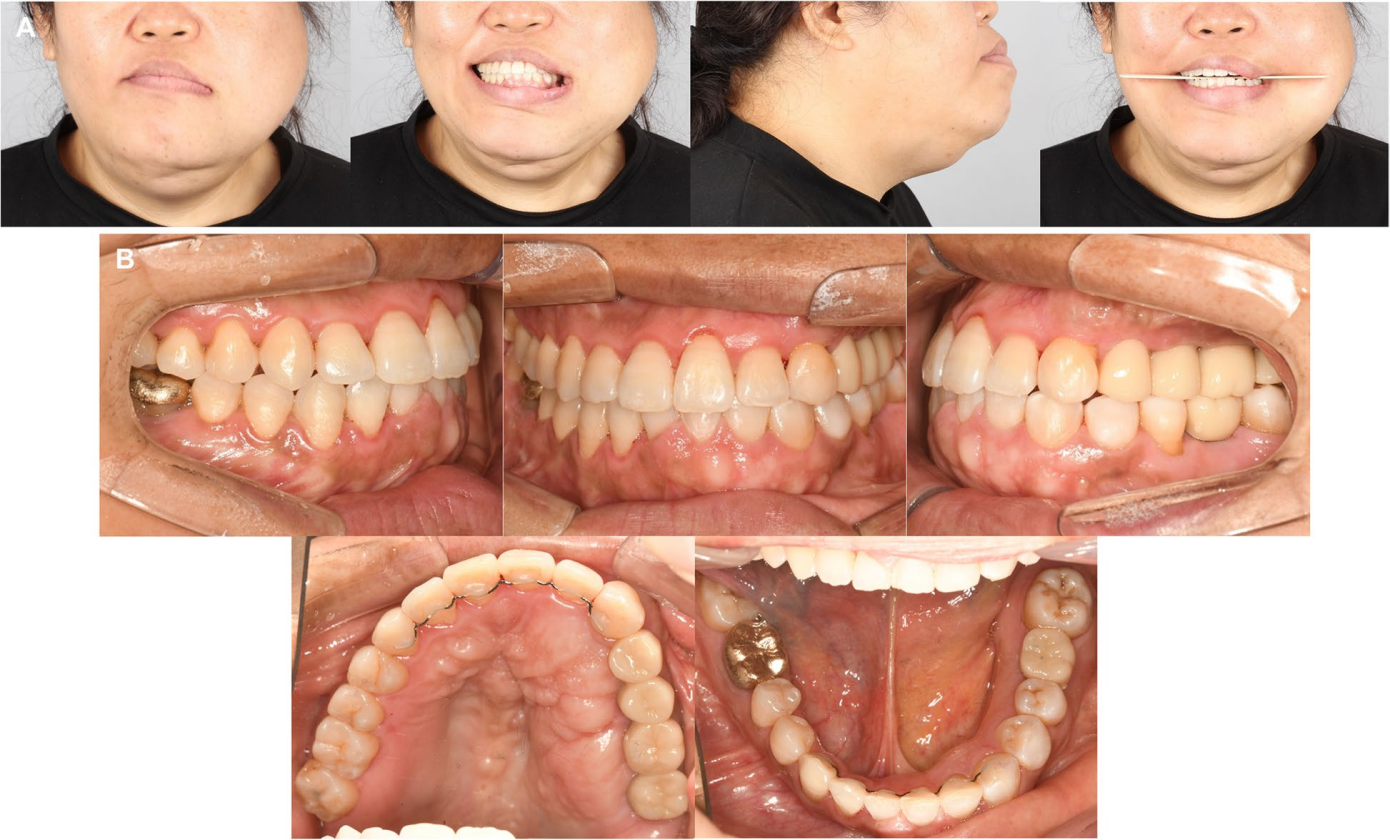
Posttreatment photographs. **A** Extraoral photographs, **B** Intraoral photographs

**Fig. 12:**
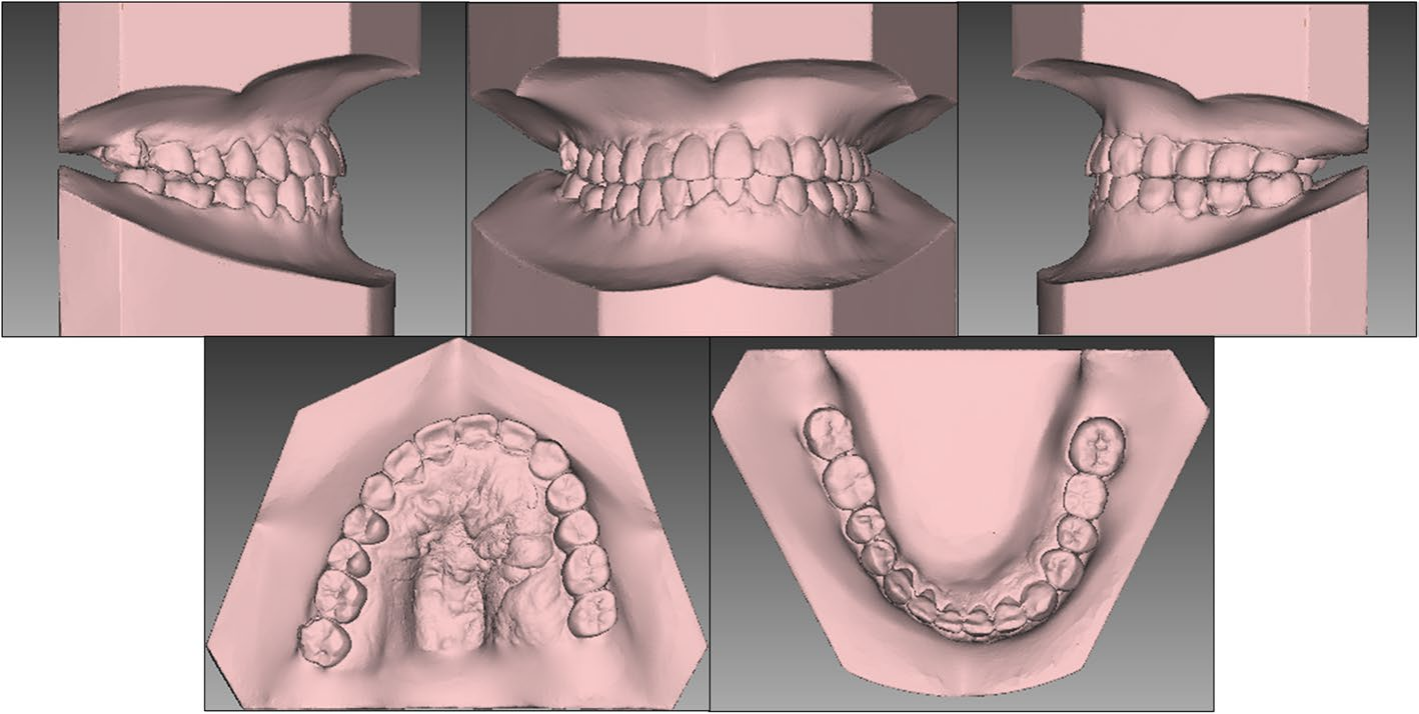
Posttreatment dental casts

At the end of the treatment, the mouth opening was maintained at 33 mm, which was well preserved from the amount secured immediately after the arthroplasty. Compared to the initial examination, the downward displacement of the left corner of the mouth has slightly improved and the caning of the occlusal plane has been resolved (Fig. 11A). Due to the limited movement of the maxillary left teeth, the maxillary arch form ended asymmetrically. Consequently, the treatment concluded with Class I canine and molar relationships on the right side and Class II relationships on the left side (Figs. 11B, 12).

**Fig. 13:**
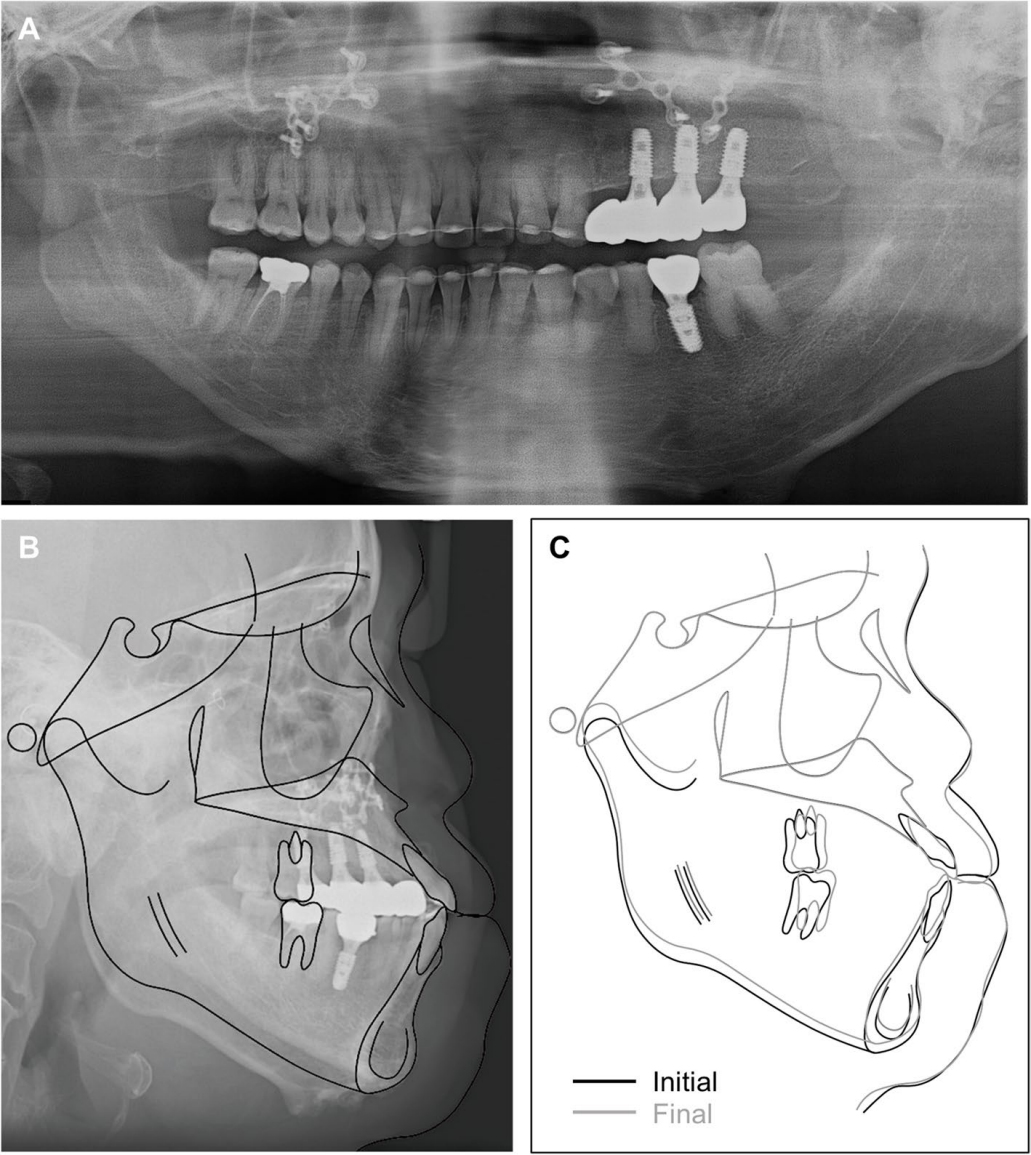
Posttreatment radiographs. **A** Panoramic radiographs, **B** Lateral cephalogram and tracing, **C** Superimposition of initial and final tracing

The patient exhibited satisfactory occlusion. A panoramic radiograph confirmed root parallelism except for the maxillary left lateral incisor (which has a curved root) and the mandibular left first premolar (Fig. 13A). The final lateral cephalogram and tracing, the superimposition of the tracings, along with the measurements, are presented in Fig. 13B, C and Table 1.

### Discussion and conclusions

In this paper, we report a rare case of true hemifacial hyperplasia accompanied by TMJ ankylosis. We aim to detail surgical approaches employed to restore mouth opening function and the sequence of procedures undertaken to recover masticatory function, including surgical intervention, orthodontic tooth movements, and prosthetic restorations.

Given that the TMJ was already ankylosed at the time of presentation, the primary objective was to separate ankylosed bones and promote mouth opening. There are several options for resolve TMJ ankylosis, such as arthroplasty, arthroplasty with joint reconstruction, total joint reconstruction, and so on [9, 10]. Regardless of the surgical approach chosen, the most critical and distressful side effect is re-ankylosis. Re-ankylosis is commonly associated with inadequate surgery, patient compliance, and biological causes [11]. Considering biological factors of this patient, bone regeneration associated with hemifacial hyperplasia could lead to re-ankylosis. The best method to determine whether the bone is still growing, in other words, to assess the lesion's growth status, is through bone Single Photon Emission Computed Tomography (SPECT) to evaluate bone metabolism. However, this procedure was not performed for this patient. In terms of adequate surgical procedure, a gap of at least 10 mm is required with interpositional material to prevent re-ankylosis. There are many options of interpositional materials such as autogenous dermal fat, buccal fat pad, fascia, full-thickness skin graft, silicon, and so on. Each method has its own advantages and disadvantages with re-ankylosis rate ranging from 0 to 32%. Thus, the choice should be based on the surgeon’s skill and judgment [9–11]. In this patient, she was informed preoperatively about the possibility of re-ankylosis due to potential postoperative bone regrowth. A silastic sheet was inserted to prevent re-ankylosis. Notably, no re-ankylosis occurred. The importance of postoperative physiotherapy in preventing re-ankylosis cannot be overstated. Early mobilization of the temporomandibular joint is critical for maintaining the surgical gap and preventing fibrous adhesion or osseous reformation. Numerous studies highlight that initiating physiotherapy within 24 to 48 h post-surgery significantly reduces the risk of re-ankylosis and promotes improved functional outcomes [12, 13]. For gap arthroplasty, creating a surgical gap of approximately 10 to 20 mm is essential to reduce the risk of re-ankylosis [14]. However, there remains a possibility of re-ankylosis due to osteoblastic activity and bone regrowth, particularly in cases with enhanced bone metabolism, as seen in hemifacial hyperplasia [15]. In this case, there were no clinical signs of additional bone growth. Importantly, the patient was followed up for a period of four years post-arthroplasty. During this time, the range of mouth opening was well maintained, demonstrating long-term stability and effectiveness of the gap arthroplasty.

While the arthroplasty resolved the TMJ ankylosis caused by hemifacial hypertrophy, reestablishing occlusion was a different challenge. The patient was presented with significantly altered occlusion and intermaxillary relationships, lacking a reference for ideal treatment goals. Therefore, we opted to sequentially eliminate causes of occlusal interference and reconstruct the occlusal relationship. After gap arthroplasty, the left maxillary tuberosity and the posterior region came into premature contact with the mandible, resulting in an overall open bite in anterior and right posterior regions. According to existing literature, hemifacial hyperplasia often involves hypertrophy of the alveolar bone on the affected side, along with macrodontia in both the maxilla and mandible [4, 6, 8]. In this case, hypertrophy of the alveolar bone was noted in the left maxilla and mandible, with excessive bone growth extending to the maxillary tuberosity. To address premature contact on the left side, several options can be considered. The first method involves performing an orthognathic surgery as reported in previous cases [16, 17]. However, this option was not considered for the patient due to a previous history of maxillary Le Fort I osteotomy with the maxilla already fixed with several plates. The second method is a segmental osteotomy of the left posterior maxillary region [18, 19]. However, bone quality and periodontal prognosis of posterior teeth were found to be poor upon flap formation. As a third option, extraction of the maxillary first molar with periodontal issues and palatal movement and intrusion of the left maxillary premolars can be considered. However, given the macrodontia of the affected teeth and the associated hypertrophy of its roots, it is anticipated that multi-directional tooth movements would require a significant amount of time. Therefore, for a shorter treatment period and quicker recovery of masticatory function, extraction of upper premolars and implant restoration were deemed more appropriate. Also, the occlusal interference caused by the proliferation of the alveolar bone itself was resolved with a maxillary osteotomy accompanied by alveoloplasty.

The bone quality or bone density of the alveolar bone proliferated due to a specific disease or on its own has not been clearly reported yet. In this patient, the hyperplastic left maxillary alveolar bone exhibited different characteristics from a normal bone, making initial stability difficult to achieve during implant placement [20, 21]. The bone quality around the maxillary first premolar was particularly poor. Thus, implants were placed in the maxillary second premolar and the first and second molar positions. The first premolar was then restored using a cantilever prosthesis, a predictable solution for restoring a partially edentulous area when there is a lack of bone to support an implant [22, 23]. Additionally, the buccopalatal width was excessively broad due to both vertical and horizontal bone hyperplasia. Thus, a surgical guide was used to place implants accurately at planned locations [24].

Gap arthroplasty can successfully alleviate trismus in patients with hemifacial hyperplasia and temporomandibular joint ankylosis. Goal-oriented surgical and orthodontic treatments were carried out based on three-dimensional prosthetic simulation. A multidisciplinary approach is essential for comprehensive occlusal rehabilitation in hemifacial hyperplasia patient.

## Data Availability

The datasets used and/or analyzed during the current study are available from the corresponding author upon reasonable request.

## Abbreviations

CBCT: Cone-beam computed tomography
TMJ: Temporomandibular joint
3D: Three-dimensional
